# Comparison of investigation methods of heat injury in grapevine (*Vitis*) and assessment to heat tolerance in different cultivars and species

**DOI:** 10.1186/1471-2229-14-156

**Published:** 2014-06-05

**Authors:** Hongguo Xu, Guojie Liu, Guotian Liu, Bofang Yan, Wei Duan, Lijun Wang, Shaohua Li

**Affiliations:** 1 College of Agronomy and Biotechnology, China Agricultural University, Beijing 100193, China; 2 Key Laboratory of Plant Resources and Beijing Key Laboratory of Grape Science and Enology, Institute of Botany, the Chinese Academy of Sciences, Beijing 100093, People's Republic of China; 3 University of Chinese Academy of Sciences, Beijing 100049, People's Republic of China; 4 Key Laboratory of Plant Germplasm Enhancement and Specialty Agriculture, Wuhan Botanical Garden, the Chinese Academy of Sciences, Wuhan 430074, People's Republic of China

## Abstract

**Background:**

In the context of global climate change, heat stress is becoming an increasingly important constraint on grapevine growth and berry quality. There is a need to breed new grape cultivars with heat tolerance and to design effective physiological defenses against heat stress. The investigation of heat injury to plants or tissues under high temperature is an important step in achieving these goals. At present, evaluation methods for heat injury include the gas exchange parameters of photosynthesis, membrane thermostability, chlorophyll content etc.; however, these methods have obvious disadvantages, such as insensitivity, inconvenience and delayed information. An effective and convenient method for investigating the heat injury of grapevine must be developed.

**Results:**

In this study, an investigation protocol for a critical temperature (47°C) and heat treatment time (40 min) was developed in detached grape leaves. Based on the results, we found that the OJIP test was superior to measuring electrolyte leakage or photosynthetic O_2_ evolution for investigating the heat injury of three cultivars of grapevine. Heat tolerance of 47 grape species and cultivars was evaluated through investigating heat injury using the OJIP test. Moreover, the electron transport chain (donor side, acceptor side and reaction center) of PSII in photosynthesis was further investigated.

**Conclusions:**

The OJIP test was a rapid, sensitive and convenient method for investigating heat injury in grapevine. An analysis of PSII function using this method indicated that the acceptor side was less sensitive to heat than was the donor side or the reaction center in grape leaves. Among the 47 taxa evaluated (cultivars, hybrids, and wild species), heat tolerance varied largely in each genotype group: most wild species and hybrids between *V. labrusca* and *V. vinifera* had relatively strong heat tolerance, but most cultivars from *V. vinifera* had relatively weak heat tolerance.

## Background

Grapevine is the most economically important fruit crop in the world, with its berries both eaten fresh and used for making wine, jam, juice, jelly, raisins and vinegar. Viticultural production is famously sensitive to climate [1-3], and temperature and moisture regimes are among the primary elements of grape terroir [3,4]. In many production regions, the maximum midday air temperature may exceed 40°C, with some regions exceeding 45°C [5-7]. High temperatures influence the development of plants and inhibit leaf photosynthesis. Exposure to high temperatures during flowering significantly inhibits berry set [8]. After fruit set, high temperatures are generally not favourable to the development secondary metabolites such as phenolic compounds [9,10] and aromatic volatiles [7]. High temperatures stimulate sugar accumulation [8], resulting in the production of wines with higher alcohol concentrations. To cope with heat stress, it is necessary to breed new cultivars with strong heat tolerance and to design effective physiological defenses against heat stress. Consequently, developing an effective and convenient method for evaluating the heat stress is a key goal.

At high temperatures, cell injury and even death may occur, which may be attributed to a catastrophic collapse of cellular organization [11]. Several physiological traits have been investigated as indicators of heat injury: gas exchange parameters of photosynthesis, including net photosynthesis rate, photosynthetic O_2_ evolution rates and stomatal conductance [12-17]; membrane thermostability, including electrolyte leakage and the content of thiobarbituric acid-reactive-substances (TBARS) [18-20]; chlorophyll content [21-23]. However, these methods all have disadvantages, including insensitivity, inconvenience in field studies and the delay of information between the initial damage and the measurable effect(s). At present, a rapid, sensitive and convenient method of investigating heat injury for evaluating heat tolerance in grapevine must be developed.

The cell membrane is thought to be a site of primary physiological injury by heat stress [24]. The injury inflicted on leaf tissues under high stress weakens the cell membrane, which leads to a leakage of electrolyte out of the cell. Thus, measuring electrolyte leakage is a common evaluation method for heat injury. Photosynthesis, which is the basis of yield and quality and has long been recognized as one of the most heat-sensitive processes in plants [11], depends on the thylakoid membrane. However, it is difficult to evaluate the heat injury for a large number of plants by measuring the net photosynthesis rate with a photosynthesis system (such as the Li-6400) or the photosynthetic O_2_ evolution rates with an oxygen electrode system due to the time required per plant. Three major heat-sensitive sites occur in the photosynthetic apparatus or process: the photosystems, mainly photosystem II (PSII), and the ATP-generating and carbon assimilation processes [25,26]. Inactivation of PSII by heat stress is related to damage of the donor side, the reaction center and the acceptor side of the photosystem’s electronic transport chain [27]. The inhibition of PSII leads to a change in variable chlorophyll *a* fluorescence, and in vivo chlorophyll may be used to detect changes in the photosynthetic apparatus [28,29]. Strasser et al. [30] developed a method (chlorophyll *a* fluorescence transient) for the analysis of the kinetics of fast fluorescence increases, using nondestructive measurements that can be taken with a high resolution of 10 μs. All oxygenic photosynthetic materials investigated to date have shown a polyphasic increase in fluorescence consisting of a sequence of phases, denoted as O, J, I and P. Therefore, the measurement of this chlorophyll *a* fluorescence transient is also called the OJIP test. The OJIP test has become a powerful tool for the in vivo investigation of PSII functioning, including its energy absorption, trapping and electron transport [28,30-33]. In crops such as wheat, cabbage and raspberry, the OJIP test has been applied in the investigation of heat injury [34-36]. However, no complete comparison study has yet been conducted between the OJIP test and traditional methods such as the measurement of electrolyte leakage and photosynthetic O_2_ evolution rates, and no information exists on the use of OJIP parameters for identifying heat tolerance in grapevine germplasm.

The aims of this study were as follow: (1) to establish a heat stress protocol for grapevine; (2) to determine which method among the OJIP test, the measurement of photosynthetic O_2_ evolution rates and electrolyte leakage was superior for assessing the heat injury of grapevines; and (3) to evaluate the heat tolerance of 47 grapevine species or cultivars through determining heat injury by the best method.

## Results

### The critical temperature (T_c_) for the investigation of heat injury of grapevines

**Figure 1:**
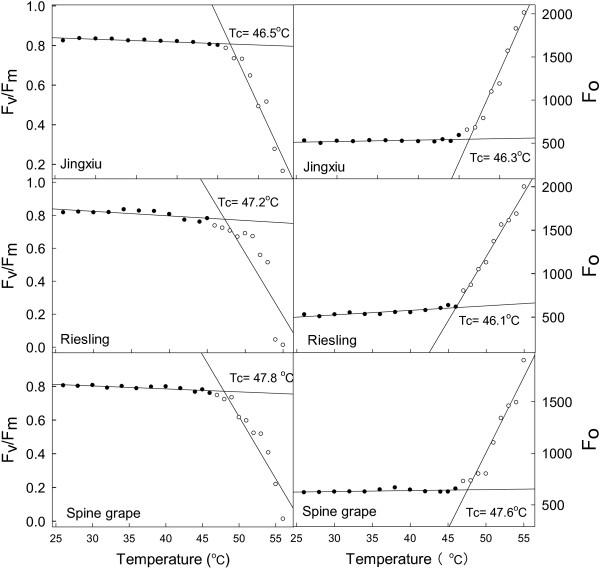
**Establishing the critical temperature (T**_**c**_**) for investigating the heat injury of grape leaves using the chlorophyll *a *fluorescence parameters F**_**v**_**/F**_**m **_**and F**_**o**_**.** T_c_ was determined from the intersection of the two regression lines extrapolated from the slow- and fast-rising portions of the temperature-dependent F_v_/F_m_ and F_o_ response.

For investigating the heat injury of grapevines, a critical temperature (T_c_) was first established. According to Weng and Lai [37], T_c_ may be determined from the intersection of the two regression lines extrapolated from the slow- and fast-rising portions of the temperature-dependent F_o_ and F_v_/F_m_ responses obtained from the OJIP test (see Method section). As shown in Figure 1, F_o_ and F_v_/F_m_ responded differently to a gradual increase of temperature in the leaves of ‘Jingxiu’, ‘Riesling’ and spine grape. These values remained relatively stable below a critical temperature, then started to increase (F_o_) or decrease (F_v_/F_m_) sharply. Little difference was observed in the critical values of ‘Jingxiu’, ‘Riesling’ and spine grape: all were approximately 47°C based on the F_v_/F_m_ (from 46.5°C to 47.8°C) and F_o_ (from 46.5°C to 47.1°C) values. Therefore, 47°C was selected as the T_c_ for evaluating the heat injury of grapevines.

### The comparison of investigation methods for heat injury of grapevines under T_c_

**Figure 2:**
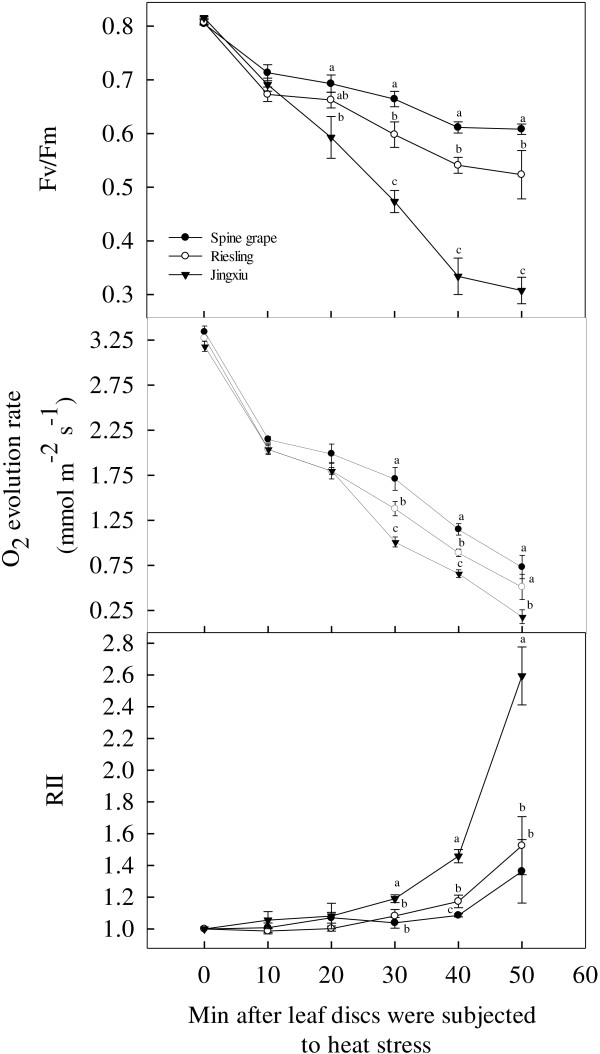
**Comparison of the three investigation methods (OJIP test, photosynthetic O**_**2 **_**evolution and electrolyte leakage) for foliar heat injury in three grape cultivars (‘Jingxiu’, ‘Riesling’ and spine grape) under the critical temperature (47°C).** F_v_/F_m_ represents the OJIP test method; RII represents the electrolyte leakage method; and the O_2_ evolution rate represents the photosynthetic O_2_ evolution method. Each value represents the mean of five replicates, and the error bars represent ± S.E.

The electrolyte leakage, photosynthetic O_2_ evolution rate and OJIP test have all been used to evaluate the heat tolerance of plants [19,20,26,38,39]. We further compared the characteristics of the above methods using the responses of leaf discs from ‘Jinxiu’, ‘Riesling’ and spine grape to heat stress at T_c_ over 50 min. In the OJIP test, we chose parameter F_v_/F_m_ to investigate heat injury. In the photosynthetic O_2_ evolution rate and electrolyte leakage methods, the O_2_ evolution rate and relative injury index (RII) (indicating the degree of injury to the cell membrane) were used as investigation parameters, respectively. Following heat stress at T_c_, the F_v_/F_m_ and O_2_ evolution rate values of the three grapevines gradually declined, while their RII values increased (Figure 2). However, the sensitivity to heat stress varied among the three cultivars. Significant differences in F_v_/F_m_ were observed among the three cultivars until after 20 min of heat stress, and significant differences in O_2_ evolution rates and RII were seen until after 30 min of stress. Significantly lower F_v_/F_m_ and O_2_ evolution rates, as well as a higher RII, were observed in ‘Jinxiu’ than in spine grape. The values of the three parameters in ‘Riesling’ fell between those of ‘Jinxiu’ and spine grape. At 40 min after the application of heat stress, the three cultivars differed significantly for F_v_/F_m_, O_2_ evolution rate and RII. Moreover, at this point, the difference among the three cultivars for F_v_/F_m_ was significantly larger than for O_2_ evolution rate or RII. At the end of the experiment, i.e., 50 min after the leaf discs were subjected to heat stress, there was a significant difference only in F_v_/F_m_ among the three cultivars. For both O_2_ evolution rate and RII, the significant differences disappeared between ‘Jingxiu’ and ‘Riesling’. In general, the heat injury of spine grape was the least, followed by the tolerances of ‘Riesling’ and ‘Jinxiu’ (Figure 2). The data indicated that 40 min is an appropriate duration of heat stress at T_c_ (47°C) for investigating the heat injury of grapevines and that the OJIP test was the most suitable among the three methods due to the sensitivity of its parameters.

### Electron transport chain of PSII in grapevines under T_c_

**Figure 3:**
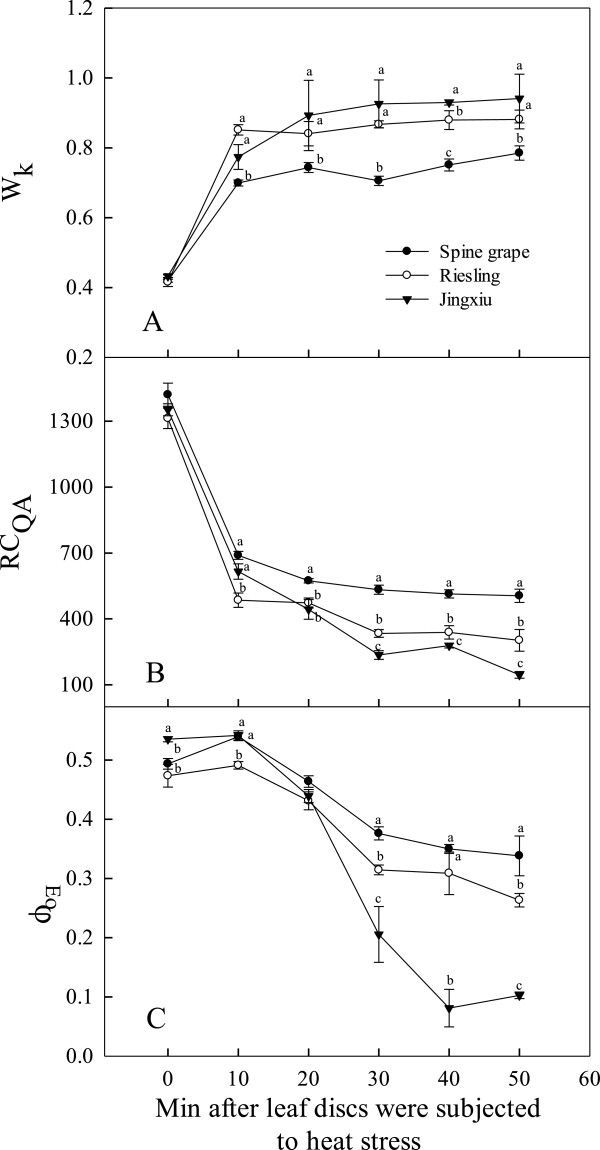
**The response of the electron transport chain of PSII, including the donor side (W**_**k**_**) (A), reaction center (RC**_**QA**_**) (B) and acceptor side (φ**_**Eo**_**) (C) parameters, of the leaves of three grape cultivars (‘Jingxiu’, ‘Riesling’ and spine grape) under the critical temperature (47°C).** Each value represents the mean of five replicates, and the error bars represent ± S.E.

The OJIP test may also reveal information regarding the electron transport chain of PSII [32]. The response of the electron transport chain of PSII to heat stress under T_c_ (47°C) was investigated using the OJIP test in ‘Jingxiu’, ‘Riesling’ and spine grape. The value of W_k_ expresses the changes of the amplitude in the K step in the OJIP test, which is used as a specific indicator of damage to the PSII donor side. In general, the W_k_ values of the three cultivars increased sharply by 10 min after the initiation of heat stress, then increased more slowly in ‘Jingxiu’ and ‘Riesling’ from 10 to 50 min over the experiment (Figure 3A). However, the W_k_ of spine grape changed little after 10 min and was significantly lower than that of the other two cultivars throughout the heat stress period. RC_QA_ indicates the density of the PSII reaction centers [40,41]. The RC_QA_ in all genotypes declined rapidly within 10 min of heat stress and continued to decrease slowly over the experiment at T_c_. The density of RC_QA_ in spine grape was significantly higher than in the other two cultivars after 10 min (Figure 3B). The changes in the quantum yield of electron transport (φ_Eo_) in the grape leaves during heat stress are shown in Figure 3C. φ_Eo_ was used as an indicator of the acceptor side of the electron transport chain of PSII [40,41]. Heat stress at 47°C altered the φ_Eo_ values in the grape leaves of all three cultivars. These values were stable after 10 min of heat stress but rapidly decreased thereafter. The spine grape and ‘Riesling’ had higher φ_Eo_ values than did ‘Jingxiu’.

### Evaluation of heat tolerance in 47 cultivars (or species) of grapevine under T_c_

Generally, heat injury under heat temperature may indirectly reflect heat tolerance in plants. The more serious heat injury, the weaker heat tolerance. In this study, for evaluating heat tolerance of 47 grape cultivars (or species), the parameter F_v_/F_m_ of the OJIP test was chosen to investigate their heat injury under T_c_. We measured the F_v_/F_m_ of these genotypes in May, June and July of 2012 and June and July of 2013. Positive correlations for the F_v_/F_m_ values in these grape leaves were observed among the different sampling times (Table 1). Our experiment was conducted in Beijing (latitude from 39°26' to 41°03', longitude from 115°25' to 117°30'), where the average daily temperature (16°C–25°C) and lower rainfall in May are more suitable for grapevine growth than are conditions in June and July. Therefore, only the data from May 2012 are reported in this paper, as shown in Table 2. The F_v_/F_m_ values varied greatly with the genetic backgrounds in each genotype group, especially in *V. vinifera*. Most wild grapevines had higher F_v_/F_m_ values than did domesticated cultivars. The highest F_v_/F_m_ value was found in *V. davidii* (1, number in the Table 2, same below) at 0.68, followed by a value of 0.62 in *V. ripara* (10). *V. rubra* (9) had the lowest F_v_/F_m_ value at only 0.39. Interspecific hybrids among wild grapevines had moderate F_v_/F_m_ values, ranging from 0.54 to 0.32 with an average value of 0.43. However, interspecific hybrids between *V. vinifera* and *V. labrusca* had relatively high F_v_/F_m_ values. The highest F_v_/F_m_ values were found in ‘Kangtai’ (18) at 0.68, followed by ‘Mitsushiru’ (19) at 0.65. ‘Jingyou’ (26) had the lowest F_v_/F_m_ value at 0.34. The F_v_/F_m_ values of the cultivars of *V. vinifera* ranged from 0.68 to 0.34, and most cultivars had lower F_v_/F_m_ values. The highest F_v_/F_m_ values were found in ‘Riesling’ (28) and ‘Cabernet Sauvignon’ (29) at 0.63 each, followed by ‘Black Balad’ at 0.61. ‘Jingyu’ (42), ‘Muscat Hamburg’ (45), ‘Cabernet Franc’ (43) and ‘Yan73’ (44) all had very low F_v_/F_m_ values of only 0.25, 0.24, 0.23 and 0.20, respectively.

**Table 1:** **Correlation analysis of the chlorophyll *a *** **fluorescence parameter F**_**v**_**/F**_**m **_**among different sampling times in grape leaves under a heat stress of 47°C for 40 min**

	**F**_**v**_**/F**_**m **_**(05/2012)**	**F**_**v**_**/F**_**m **_**(06/2012)**	**F**_**v**_**/F**_**m **_**(07/2012)**	**F**_**v**_**/F**_**m **_**(06/2013)**	**F**_**v**_**/F**_**m **_**(07/2013)**
F_v_/F_m_ (05/2012)	1	0.834**	0.623**	0.542**	0.602**
F_v_/F_m_ (06/2012)		1	0.695**	0.683**	0.580**
F_v_/F_m_ (07/2012)			1	0.640**	0.411**
F_v_/F_m_ (06/2013)				1	0.480**
F_v_/F_m_ (07/2013)					1

The asterisks * and ** indicate a significant correlation at *P* < 0.05 and *P* < 0.01, respectively.

**Table 2:** **Heat tolerance of grape cultivars or species evaluated using the chlorophyll a fluorescence parameter** F_v_/F_m_

**No**	**Cultivars or species**	**F**_**v**_**/F**_**m**_	**Germplasm group**	**Average F**_**v**_**/F**_**m **_**in a group**
1	*V. davidii*	0.68 ± 0.01a	Wild grape	0.51 ± 0.03a
2	*V. amurensis*	0.55 ± 0.02bc		
3	*V. pseudoreticulata*	0.41 ± 0.04e		
4	*V. flexuosa*	0.40 ± 0.02e		
5	*V. bryoniaefolia*	0.52 ± 0.04 cd		
6	*Shuangqing (V. amurensis*)	0.58 ± 0.01bc		
7	*V. cinerea*	0.55 ± 0.02bc		
8	*V. aestivalis*	0.46 ± 0.03de		
9	*V. rubra*	0.39 ± 0.03e		
10	*V. ripara*	0.62 ± 0.01ab		
11	5BB (*V. berlandier* × *V. ripara*)	0.33 ± 0.02c	Hybrids among wild grape	0.43 ± 0.03ab
12	5C (*V. berlandier* × *V. ripara*)	0.53 ± 0.04a		
13	SO4 (*V. berlandier* × *V. ripara*)	0.50 ± 0.03ab		
14	Beichun (*V. vinifera* × *V. amurensis*)	0.41 ± 0.03bc		
15	Beihong (*V. vinifera* × *V. amurensis*)	0.32 ± 0.02c		
16	*Beifeng (V. vinifera* × *V. adstricta*)	0.48 ± 0.01ab		
17	Beta (*V. labrusca* × *V. ripara*)	0.44 ± 0.05ab		
18	Kangtai	0.68 ± 0.01a	Hybrids between *V. vinifera* and *V. labrusca*	0.50 ± 0.04a
19	Mitsushiru	0.65 ± 0.01a		
20	Kyoho	0.55 ± 0.03b		
21	Takasumi	0.50 ± 0.02bc		
22	Gaoqi	0.49 ± 0.02bc		
23	Izunishiki	0.46 ± 0.04c		
24	Jingya	0.44 ± 0.03c		
25	Fujiminori Grape	0.35 ± 0.04d		
26	Jingyou	0.34 ± 0.02d		
27	Parasaurolophus	0.56 ± 0.02b		
28	Riesling	0.63 ± 0.02a		
29	Cabernet Sauvignon	0.63 ± 0.01a		
30	Black balad	0.61 ± 0.03a		
31	Red balad	0.51 ± 0.02b		
32	Chardonnay	0.48 ± 0.04bc		
33	Ruby Seedless	0.42 ± 0.03def		
34	Alexander	0.39 ± 0.04cde		
35	Xiangfei	0.37 ± 0.02def		
36	Jingxiangyu	0.36 ± 0.03def		
37	Italian Riesling	0.34 ± 0.07efg		
38	Red Globe	0.34 ± 0.01efg	*V. vinifera*	0.38 ± 0.03b
39	Merlot	0.33 ± 0.01efg		
40	Cardinal	0.28 ± 0.02fgh		
41	Gros Colman	0.28 ± 0.03gh		
42	Jingyu	0.25 ± 0.01gh		
43	Cabernet Franc	0.23 ± 0.02 h		
44	Yan73	0.19 ± 0.02 h		
45	Muscat	0.24 ± 0.02 h		
46	Nilawa	0.25 ± 0.01gh		
47	Jingyan	0.45 ± 0.02bcd		

Values are means ± S.E; Different letters indicate means are significantly different at *P* < 0.05.

To reveal the relationship between the electron transport chain of PSII and the heat tolrance of grape leaves, the F_v_/F_m_, W_k_, RC_QA_ and φ_Eo_ of the 47 grape cultivars (or species) were further analyzed using correlation analysis based on the data from May 2012. Table 3 shows that F_v_/F_m_ was positively correlated with RC_QA_ and φ_Eo_ but negative correlated with W_k_, indicating that higher F_v_/F_m_ values were associated with higher RC_QA_ and φ_Eo_ values but lower W_k_ values. These results suggest that the heat tolerance of grapevine is associated with the electron transport chain, including the donor side, reaction center and acceptor side of PSII.

**Table 3:** **Correlation analysis among F**_**v**_**/F**_**m**_**, W**_**k**_**, RC**_**QA **_**and φ**_**Eo**_

	**F**_**v**_**/F**_**m**_	**RC**_**QA**_	**φ**_**Eo**_	**W**_**k**_
F_v_/F_m_	1.00	0.84^**^	0.79^**^	-0.41^**^
RC_QA_		1.00	0.49^**^	-0.73^**^
φ_Eo_			1.00	-0.03
W_k_				1.00

The asterisks * and ** indicate a significant correlation at *P* < 0.05 and *P* < 0.01, respectively.

## Discussion

### Methods of investigating heat injury in grapevines

As determined by Weng and Lai [37], T_c_ was easily calculated as 47°C using the OJIP test parameters of F_o_ and F_v_/F_m._. Based on this result, the heat injury of ‘Jingxiu’, ‘Reisling’ and spine grape was investigated using three methods (OJIP test, photosynthetic O_2_ evolution rates and electrolyte leakage). These methods led to the same conclusion: the heat injury of spine grape was the least, followed by ‘Riesling’ and ‘Jinxiu’. Although the three methods obtained the same results, they exhibited different characteristics. First, the processes of measuring electrolyte leakage and photosynthetic O_2_ evolution rates were more complex and required more time than the OJIP test (see Methods section for details). Second, the measurement of electrolyte leakage and photosynthetic O_2_ evolution rates must be conducted in the lab and requires small leaf discs. The former method requires a conductivity meter and a water bath, while the later requires an oxygen electrode system, a computer and a water bath. The OJIP test can be conducted in the lab or the field, and either leaf discs or whole leaves may be measured using the Handy Plant Efficiency Analyzer (volume: 175 × 80 × 40 mm^3^; Weight: 0.65 Kg). Third, measuring electrolyte leakage or photosynthetic O_2_ evolution rates yields only a single parameter, but the OJIP test can produce several parameters, including information regarding the electron transport chain of the photosynthetic apparatus. Fourth, the sensitivity of the three methods differed. As shown in Figure 2, significant differences in the F_v_/F_m_ and O_2_ evolution rate values among ‘Jinxiu’, ‘Riesling’ and spine grape appeared after 30 min of heat stress at T_c_, but differences in the RII values appeared only after 40 min. After 50 min of heat stress at T_c_, the differences in O_2_ evolution rate and RII values between ‘Jinxiu’ and ‘Riesling’ disappeared, but the differences of F_v_/F_m_ among the three cultivars remained. In general, the OJIP test was a rapid, sensitive and convenient method for measuring heat injury in grapevine. Moreover, the reproducibility of the method is very high, as shown in the correlation analysis between different years and different months (Table 1). Additionally, the Handy Plant Efficiency Analyzer may be used directly in the field. However, this evaluation relies primarily on photosynthesis and does not consider other physiological processes. The results of this study may be further applied in molecular breeding and quantitative trait analysis (QTL) by providing stable, sensitive phenotypic data for heat injury.

### Heat injury in grape leaves is related to the photosynthetic electron transport chain of PSII

Photosynthesis, especially the electron transport chain of PSII, is highly sensitive to high-temperature stress [42,43]. However, it is difficult to pinpoint the specific limiting steps that control the temperature response of the electron transport chain [44]. In our study, the decrease of the photosynthetic O_2_ evolution rate under heat stress was associated with electron transport capacity, which showed that the PSII of the photosynthetic apparatus was damaged. The different sensitivities of the parameters derived from the OJIP test may reflect the heterogeneous behavior of PSII under heat stress conditions. W_k_ expresses the K-step in the OJIP test, which is used as a specific indicator of damage to the PSII donor side related to the oxygen evolving complex (OEC) during heat stress. In this study, the W_K_ value increased significantly by 10 min in all grape genotypes during the heat treatment, demonstrating that the OEC is one of the most vulnerable complexes of the photosynthetic electron transport chain. The results also showed that the stability of the OEC differs among genotypes, as the OECs of ‘Jingxiu’ and ‘Riesling’ were more vulnerable than those of the other genotypes.

The density of RC_QA_ may reflect the density of Q_A_-reducing PSII reaction centers [41], and the PSII reaction center is also one of the sites damaged by heat stress [45]. In our study, during heat stress at 47°C, the density of RC_QA_ decreased rapidly by 10 min for all genotypes, which indicated that the PSII reaction center was sensitive to heat and that the thermostability of the PSII reaction center differed among cultivars. The parameter φ_Eo_ represents the quantum yield or the energy distribution ratio of the acceptor side of PSII. The decrease in φ_Eo_ showed that the activity of electron transport beyond Q_A_ was inhibited in grape leaves after 50 min of heat stress, but after only 10 min, the φ_Eo_ values showed almost no change. These results indicated that while heat stress damaged the acceptor side of PSII, this structure was relatively stable in the initial stages of heat stress. The correlation analysis of the evaluation of different cultivars (species) further corroborated these results (Table 3). Therefore, the OJIP test can also reveal the relationship between heat injury in grape leaves and the photosynthetic electron transport chain of PSII.

## Conclusions

The OJIP test was quicker, more sensitive and more convenient for investigating the heat injury of grape leaves than were measurements of photosynthetic O_2_ evolution rates or electrolyte leakage. Moreover, PSII functional analysis using the OJIP test indicated that the acceptor side of the photosystem II was less damaged by heat than were the donor side or the reaction center in grape leaves. The heat tolerance of 47 cultivars (or species) was evaluated by determining heat injury using this method. In general, the heat tolerance among cultivars or species varied largely in each genotype group. Most wild species and some hybrids of *V. labrusca* and *V. vinifera* had relatively strong heat tolerance, while most cultivars of *V. vinifera* had relatively weak heat tolerance.

## Methods

### Plant materials

A total of 47 wild species and cultivars were used in this study (Table 4). All of the grapevines were planted at the Germplasm Repository for Grapevines in the Institute of Botany of the Chinese Academy of Sciences, located in Beijing, in the spring of 1993. The vines, trained to bilateral cordons, were spaced 1.5 m apart within the row and 2.5 m apart between the rows with a north-south row orientation. All vines were subjected to similar management practices for irrigation, fertilization, soil management, pruning, and disease control. Healthy leaves of approximately 30 days in age were used in this study. In May, June and July of 2012 and June and July of 2013, samples were taken in the morning, placed in the dark with the petiole in water, and then treated by heat stress.

**Table 4:** Grape cultivars or species used in this study

**Germplasm groups**	**Cultivar number**	**Cultivars**
Wild grape	10	*V. davidii* (1), *V. amurensis* (2), *V. pseudoreticulata* (3), *V. flexuosa* (4), *V. bryoniaefolia* (5), Shuangqing (*V. amurensis*, 6), *V. cinerea* (7), *V. aestivalis* (8), *V. rubra* (9), *V. ripara* (10)
Hybrids among wild grape	7	5BB (*V. berlandier* × *V. ripara*) (11), 5C (*V. berlandier* × *V. ripara*) (12), SO4 (*V. berlandier* × *V. ripara*) (13), Beichun (*V. vinifera* × *V. amurensis*) (14), Beihong (*V. vinifera* × *V. amurensis*) (15), Beifeng (*V. vinifera* × *V. adstricta*) (16), Beta (*V. labrusca* × V. *ripara*) (17)
Hybrids between *V. vinifera* and *V. labrusca*	10	Kangtai (18), Mitsushiru (19), Kyoho (20), Takasumi
		(21), Gaoqi (22), Izunishiki (23), Jingya (24), Fujiminori Grape (25), Jingyou (26), Parasaurolophus (27)
*V. vinifera*	20	Riesling (28), Cabernet Sauvignon (29), Black balad (30), Red balad (31), Chardonnay (32), Ruby Seedless (33), Alexander (34), Xiangfei (35), Jingxiangyu (36), Red Globe (37), Italian Riesling (38), Merlot (39), Cardinal (40), Gros Colman (41), Jingyu (42), Cabernet Franc (43), Yan73 (44), Muscat Hamburg (45), Nilawa (46), Jingyan (47)

### Heat stress process, critical temperature and appropriate heat stress time

The heat stress process was as follows: leaf discs (5.5 cm in diameter) were cut from the detached sample leaves, wrapped in a wet paper towel and placed in a small vessel made of aluminum foil. The vessels were then floated on water in a temperature-controlled water bath. To compare the effects of different evaluation methods for heat injury and to evaluate heat tolerance in the different species and cultivars, the critical temperature (T_c_) and appropriate heat stress time were first determined. According to the methods of Weng and Lai [37], T_c_ is the temperature at which the chlorophyll *a* fluorescence parameter F_o_ starts to increase sharply or F_v_/F_m_ decreases sharply. The experiment was conducted in three cultivars or species: ‘Jingxiu’ (*V. vinifera*), ‘Riesling’ (*V. vinifera*) and spine grape (*V. davidii*) in May of 2012. Leaf discs of each cultivar were heated from 25°C to 55°C at a rate of approximately 1°C min^-1^ in darkness, according to the above heat stress process. F_v_/F_m_ and F_o_ were measured every 1–2 min using a Handy Plant Efficiency Analyzer (Hansatech Instruments, King’s Lynn, Norfolk, UK) (details shown below). T_c_ was determined from the intersection of the two regression lines extrapolated from the slow- and fast-rising portions of the temperature-dependent fluorescence parameter F_o_ or F_v_/F_m_ responses. To determine the appropriate heat stress duration, the leaf discs were exposed to T_c_ for 50 min, and the F_v_/F_m_, electrolyte leakage and photosynthetic O_2_ evolution rates were determined every 10 min. The time at which a significant difference for each parameter was observed among the three cultivars was regarded as the appropriate heat stress time for the study.

### Three methods of investigating heat injury (electrolyte leakage, photosynthetic O_2_ evolution rate, chlorophyll *a* fluorescence)

After determining the critical temperature and appropriate heat stress time, the three investigating methods, electrolyte leakage, photosynthetic O_2_ evolution rate and the OJIP test, were compared in May of 2012.

To measure electrolyte leakage, the heat-stressed leaf discs (5.5 cm in diameter) were again cut into smaller leaf discs (1 cm in diameter) and washed with deionized water, then incubated in 10 ml of deionized water at 25°C for 6 h using a shaker. The initial electrical conductivity (E_1_) was read using a FE30 conductivity meter (Mettler Toledo, Shanghai, China). The samples were then boiled at 95°C for 60 min and cooled to 25°C before being measured again for electrical conductivity (E_2_). The relative electrolyte leakage (REL) was estimated using the following formula: REL (%) = E_1_/E_2_ × 100. The relative injury to cell membranes after heat stress treatment (47°C) was calculated using the following formula: RII (relative injury index) = T_REL_/C_REL_, where T and C refer to the heat stress (47°C) and control (25°C) temperatures, respectively [14].

The photosynthetic O_2_ evolution rates of the leaf discs were measured using a ChloroLab-2 liquid-phase oxygen electrode system (Hansatech Instruments, King’s Lynn, Norfolk, UK), as described previously [46]. The heat-treated leaf discs (5.5 cm in diameter) were first adapted at 25°C in the dark for 30 min, then cut into smaller leaf discs (1 cm in diameter) that were immediately placed into a reaction chamber filled with 1.5 ml 50 mM Hepes-KOH (pH 7.2), 0.5 mM CaSO_4_ and 20 mM NaHCO_3_. At the same time, the leaf discs were exposed to a photon flux density of 800 μmol m^-2^ s^-1^, which was provided by an array of light-emitting diodes. After 10 min of equilibration under this light, the O_2_ evolution was measured, and the data were continuously monitored for 10 min. The O_2_ evolution rate was calculated over the last 3 min of measurement [47].

The OJIP test was conducted using a Handy Plant Efficiency Analyzer after the heat-stressed leaf discs had been adapted at 25°C for 30 min in the dark. The OJIP test was performed under a saturating photon flux density of 3000 μmol m^-2^ s^-1^ provided by an array of three light-emitting diodes (peak 650 nm). The fluorescence signals were recorded within a time span from 10 μs to 1 s, with a data acquisition rate of 10 μs for the first 2 ms and every 1 ms thereafter. The following data from the original measurements were used: F_k_: the fluorescence intensity at 300 μs [required for the calculation of the initial slope (M) of the relative variable fluorescence (V) kinetics and W_k_]; F_j_: the fluorescence intensity at 2 ms (the J-step); F_i_: the fluorescence intensity at 30 ms (the I-step); and F_m_: the maximal fluorescence intensity (the P-step). The derived parameters were as follows: F_o_, the fluorescence intensity at 50 μs; W_k_, calculated as W_k_ = (F_k_ - F_o_)/(F_j_ - F_o_) and assumed to represents the damage to the oxygen evolving complex (OEC) of PSII; and RC_QA_, calculated as the number of active PSII RCs per cross section (CS) at t = t_m_ using the formula RC_QA_ = RC/CS_m_ = φ_Po_ × (V_j_/M_o_) × (ABS/CS_m_) and assumed to represent the density of Q_A_-reducing reaction centers (RCs). Here, ABS represents the total photon flux absorbed by the PSII antenna pigments. According to the energy flux theory proposed by Strasser et al. [47], the total ABS is partially trapped by the PSII RCs, and the fraction of the ABS used to reduce Q_A_ is labeled as TR, whereas the electron transport flux from Q_A_ to Q_B_ is labeled as ET. The yield indices or flux ratios can then be derived as follows: the parameter φ_Po_, representing the maximum quantum yield of primary photochemistry, is calculated as the ratio of TR/ABS at t = 0 using the equation φ_Po_ = TR_o_/ABS = 1 – F_o_/F_m_ = F_v_/F_m_; the parameter *φ*_Eo_, representing the quantum yield of the electron transport flux from Q_A_ to Q_B_ (at t = 0), is calculated using the equation φ_Eo_ = ET_o_/ABS = (F_m_ - F_j_)/F_m_. All of these parameters are shown in Table 5.

**Table 5:** Summary of parameters, formulae and their descriptions using data extracted from the OJIP test

**Fluorescence parameters**	**Fluorescence parameters description**
Extracted parameters	
F_t_	Fluorescence intensity at time t after onset of actinic illumination
F_50 μs_	Minimum reliable recorded fluorescence at 50 μs with the Handy PEA
F_k_ (F_300 μs_)	Fluorescence intensity at 300 μs
F_P_	Maximum recorded (=maximum possible) fluorescence at P-step
Area	Total complementary area between fluorescence induction curve and F = *F*_m_
Derived parameters	
F_o_ ≌ F_50 μs_	Minimum fluorescence, when all PSII RCs are open
F_m_ = F_P_	Maximum fluorescence, when all PSII RCs are closed
V_j_ = (F_j_ - F_o_)/(F_m_ - F_o_)	Relative variable fluorescence at the J-step (2 ms)
V_i_ = (F_i_ - F_o_)/(F_m_ - F_o_)	Relative variable fluorescence at the I-step (30 ms)
W_K_ = (F_k_ - F_o_)/(F_j_ - F_o_)	Representing the damage to oxygen evolving complex (OEC)
M_o_ = 4 (F_k_ - F_o_)/(F_m_ - F_o_)	Approximated initial increment (in ms-1) of the relative variable fluorescence
F_v_/F_m_ = 1- (F_o_/F_m_)	Maximum quantum yield of primary photochemistry at t = 0
φ_Eo_ = ET_o_/ABS = (F_m_-F_j_)/F_m_	Quantum yield for electron transport at t = 0
RC_QA_ = φ_Po_ × (ABS/CS_m_) × (V_j_/M_o_)	Amount of active PSII RCs (Q_A_-reducing PSII reaction centers) per CS at t = m

### Evaluation of heat tolerance in different grape cultivars and species using OJIP test

The heat tolerance of the leaves of 47 grape cultivars (or species) were evaluated in May, June and July of 2012 and June and July of 2013 based on the above heat stress procedures. After the leaf discs were exposed to high temperatures (47°C) for 40 min, the OJIP test was performed using a Handy Plant Efficiency Analyzer to investigate heat injury which indirectly reflects heat tolerance.

### Statistical analysis

The data were processed using SPSS 13.0 for Windows, and each value of the means and standard errors in the figures represents five replications. Differences were considered significant at a probability level of *P* < 0.05 according to Duncan’s multiple range comparison.

## Competing interests

The authors declare that they have no competing interests.

## Authors’ contributions

XHG performed the experiments and wrote the manuscript. LGJ designed the experiment and reviewed the manuscript. WLJ designed the experiments and wrote the manuscript. LGT and YBF helped perform the experiments. DW helped design the experiment. LSH designed the experiment and reviewed the manuscript. All authors have read and approved the final manuscript.

## Authors’ information

Hongguo Xu and Guojie Liu: Co-first author.
